# Supplementary material for: Mechanistic insights into the non-equilibrium thermodynamics of nitrogen fixation via acoustic cavitation
Source: Nat Commun. 2026 Feb 12;17:2682. doi: 10.1038/s41467-026-69466-1 (PMC13009208; doi:10.1038/s41467-026-69466-1)
Supplement: Supplementary file 1 — Supplementary information [file 41467_2026_69466_MOESM1_ESM.pdf]

Supplementary information for

Mechanistic insights into the non-equilibrium thermodynamics of nitrogen fixation via acoustic cavitation

Xuelei Pan[1,†], Davide Bernardo Preso[1,†], Qian Liu[2,†], Lucia Mullings[1], Mohamad Salimi[1], Yi Qin[1], Pankaj S. Sinhmar[1], James Kwan[1,*]

[1] Department of Engineering Science, University of Oxford, Parks Road, Oxford OX1 3PJ, United Kingdom

[2] Hoffmann Institute of Advanced Materials, Shenzhen Polytechnic University, 7098 Liuxian Boulevard, Shenzhen 518055, China

[†] These authors contributed equally: Xuelei Pan, Davide Bernardo Preso, and Qian Liu.

[*]Corresponding author, email: james.kwan@balliol.ox.ac.uk

Supplementary notes

Supplementary Note 1 - The calculation of duty cycle and effective reaction time

In typical experiments, the ultrasound is set to burst modes. The duty cycle D (%) can be calculated by $D = \frac{100n}{fT}$, where n is the number of cycles, f is the frequency, and T is the burst period. The effective reaction time T_e is calculated by $T_e = TD$, where T is the total time. If considering the total reaction time, including the ultrasound off time, the nominal power can be used to describe the average input power. The nominal power P_n is calculated by $P_n = PD$, where P is the electrical energy.

Supplementary Note 2 - The cavitation probability calculation

The experimental setup and parameters are described in Methods section in the main manuscript. For each burst, a 5000-sample window corresponding to the active insonation period was isolated and its single-sided power spectral density (PSD) estimated using Welch's method (MATLAB function pwelch). A 2048-sample Hanning window with 1024-sample overlap and a 4096-point fast Fourier transform (FFT) was used. Broadband acoustic emissions were quantified by integrating PSD with respect to frequency from 2 MHz to 10 MHz, after excluding ± 50 kHz frequency bins around the fundamental driving frequency and its first six harmonics. The integrated broadband energy E_{bb} for each burst was computed as:

$$E_{\mathrm{bb}} = \sum_{2 \le f \le 10\,\mathrm{MHz}} \mathrm{PSD}(f) \cdot M(f) \cdot \Delta f \tag{1}$$

where Δf is the frequency resolution, and $M(f)$ is a binary mask equal to zero within the excluded harmonic frequency bins and unity elsewhere.

Cavitation probability was determined following a procedure adapted from Haller et al.[1]. For each medium and excitation frequency, baseline broadband-energy statistics were first established under non-cavitation conditions (e.g. peak negative pressure < 1.5 MPa), yielding a baseline mean broadband energy, μ_0, and its standard deviation σ_0. At higher pressures, each burst was classified into events defined as 'positive', 'negative' or 'unclear', by comparing the broadband energy deviation from the baseline:

$$\mathrm{event(burst)} = \begin{cases} \text{positive, if } |E_{\mathrm{bb}} - \mu_0| > 5 \cdot \sigma_0 \\ \text{negative, if } |E_{\mathrm{bb}} - \mu_0| < 3 \cdot \sigma_0 \\ \text{unclear otherwise} \end{cases} \tag{2}$$

The number of positive N_{pos}, negative N_{neg}, and unclear N_{unc} events were summed across N_{burst}, for each sample, at each pressure. Omitting the unclear events, P_{cav} was then defined as:

$$P_{\mathrm{cav}} = \frac{N_{\mathrm{pos}}}{N_{\mathrm{pos}} + N_{\mathrm{neg}}} \text{ or equivalently} \frac{N_{\mathrm{pos}}}{N_{\mathrm{bursts}} - N_{\mathrm{unc}}} \tag{3}$$

To characterise the pressure dependence of cavitation probability, P_{cav} was modelled using a sigmoidal function. For each medium and excitation frequency, P_{cav} as a function of peak negative pressure was fitted using non-linear least-squares regressing in MATLAB with a logistic function of the form:

$$P(x) = \frac{1}{1 + e^{[-A(x - x_0)]}} \tag{4}$$

where x is the peak negative pressure, A describes the steepness of the transition, and x_0 corresponds to the pressure at which $P_{\mathrm{cav}} = 0.5$.

Supplementary Note 3 - Estimation of acoustic potential energy

1. Pressure field measurement and spatial scanning

The experimental setup is described in Methods section in the main manuscript. At each spatial position in the scanned 3D grid, the hydrophone recorded a full time-domain voltage signal $v(\mathbf{r}, t)$ (V) across the pulse

duration, sampled at 25 MHz on an oscilloscope (LeCroy, LT264). In post-processing, $v(\mathbf{r},t)$ was converted to an acoustic pressure $p(\mathbf{r},t)$ (MPa) using an interpolated FOH sensitivity (V MPa^{-1}) at driving frequency, f (Hz), where:

$$p(\mathbf{r},t) = \frac{v(\mathbf{r},t)}{S(f)} \tag{5}$$

Each waveform also included a pre-sampling window (before ultrasound arrival) and post-sampling window (after the field had passed) to define baseline noise. Only the first propagation of ultrasound through the test tube wall was considered (i.e. neglecting any reflections), as following signal peaks dropped by at least −6dB in energy density. Scan measurements were performed whilst driving the transducer at low electrical power to avoid non-linear effects, protect the hydrophone, and prevent signal saturation of the pre-amplifier system.

2. Noise modelling and signal windowing

Raw time-domain waveforms showed low-amplitude baseline noise before the acoustic burst. To eliminate noise bias, a Gaussian distribution was fit to the pre-signal region, as shown by the Gaussian overlay in Supplementary Fig. 25. The first 50 waveform samples (equating to 2μs) at each point in the 3D space were used to calculate the overall mean, μ_{noise} (MPa), and standard deviation, σ_{noise} (MPa), of the noise. Only pressure values with magnitude exceeding $\mu_{\text{noise}} + 3 \cdot \sigma_{\text{noise}}$ were retained for energy density calculation, to give a 99.7% confidence level. The difference between the first and last time point where this occurs is denoted pulse window length τ (s).

3. Calculation of instantaneous acoustic energy density

In acoustic wave propagation, the instantaneous acoustic energy density $E(\mathbf{r},t)$ at a point in space $\mathbf{r}$, and time t is the summation of the potential and kinetic energy densities:

$$E(\mathbf{r},t) = E_p(\mathbf{r},t) + E_k(\mathbf{r},t) \tag{6}$$

with:

$$E_p(\mathbf{r},t) = \frac{1}{2}\frac{p(\mathbf{r},t)^2}{\rho c^2} \,(\text{J m}^{-3}) \tag{7}$$

$$E_k(\mathbf{r},t) = \frac{1}{2}\rho \mathbf{u}\cdot\mathbf{u} \,(\text{J m}^{-3}) \tag{8}$$

where $\mathbf{u}(\mathbf{r},t)$ is the particle velocity vector (m s^{-1}), ρ is the fluid density (kg m^{-3}), and c is the speed of sound in the fluid (m s^{-1}).

However, without direct measurement of $\mathbf{u}$, or a plane-wave assumption to estimate the velocity, a potential energy density approximation is used. Theoretical and numerical studies of acoustic cavitation consistently find that compressive pressure-force work is the dominant source of energy input to collapsing bubbles[2-4]. Sonochemistry relies on the radical formation initiated by acoustic cavitation, so setting $E \approx E_{\text{p}}$, is a reasonable assumption.

For pulsed ultrasound signals, the average potential energy density over a single pulse is defined as:

$$\bar{E}_{\text{PA}}(\mathbf{r}) = \frac{1}{\tau}\int_0^\tau E(\mathbf{r},t)\,\text{d}t = \frac{1}{\tau}\int_0^\tau \frac{1}{2}\cdot\frac{p(\mathbf{r},t)^2}{\rho c^2}\text{d}t \,(\text{J m}^{-3}) \tag{9}$$

To account for how energy is delivered over time (e.g., over an experimental or pulsing schedule), a temporal average energy density can also be defined by scaling $\bar{E}_{\text{PA}}$ using the duty cycle, D:

$$\bar{E}_{\text{TA}}(\mathbf{r}) = D \cdot \bar{E}_{\text{PA}}(\mathbf{r}) \tag{10}$$

where T_B is the burst period (s), equal to the time between consecutive ultrasound pulse emission.

The total acoustic energy transferred into an arbitrary volume V (m^3) over an experimental duration T_{exp} (s) is estimated by integrating $\bar{E}_{\text{TA}}(\mathbf{r})$ spatially over V and scaling by the total number of pulses endured, N_{pulse}:

$$E_{\text{in}} = \iiint_V \bar{E}_{\text{TA}}(\mathbf{r})\, \mathrm{d}V \cdot N_{\text{pulse}} (J) \tag{11}$$

$$N_{\text{pulse}} = \frac{T_{\text{exp}}}{T_{\text{B}}} \tag{12}$$

Thus, combining with equation (10) results in a total energy of:

$$E_{\text{in}} = \iiint_V \bar{E}_{\text{PA}}(\mathbf{r})\, \mathrm{d}V \cdot D \cdot \left(\frac{T_{\text{exp}}}{T_{\text{B}}}\right) (J) \tag{13}$$

4. Spatial thresholding –6dB volume

The focal volume used to integrate $\bar{E}_{\text{PA}}(\mathbf{r})$ was defined using a –6 dB criterion relative to the maximum average energy density $\bar{E}_{\text{PA,max}}$, across the space (denoted by the focal point $\mathbf{r}_0$). This is intended to emulate the –6 dB beam volume commonly used to characterise the 'active' region of diagnostic ultrasonic transducers[5]. The –6 dB mask is constructed as so:

$$V_{-6\text{dB}} = \left\{\mathbf{r} \in V \middle| \bar{E}_{\text{PA}}(\mathbf{r}) \geq 0.25 \cdot \bar{E}_{\text{PA,max}}\right\} \tag{14}$$

The total volume spanned by the –6 dB region is also calculated. Supplementary Fig. 26 shows an example of the spatial distribution of the maximum energy density achieved over burst duration within the sample, as well as the estimated contour.

5. Discrete data total energy calculation

For implementation, the integral in equation (13) can be rewritten in a discrete form. Using timestep Δt, the average pulse energy density is approximated as:

$$\bar{E}_{\text{PA}}(\mathbf{r}) = \frac{1}{\tau}\sum_{i=1}^{N_t}\left(\frac{1}{2}\cdot\frac{p(\mathbf{r}, t_i)^2}{\rho c^2}\right)\Delta t \tag{15}$$

Then summing and scaling over the –6dB volume (with scanned voxel increment $\Delta V = \Delta x \cdot \Delta y \cdot \Delta z$) gives:

$$E_{\text{in}} = \left(\sum_{\mathbf{r}\in V_{-6\text{dB}}} \bar{E}_{\text{PA}}(\mathbf{r}) \cdot \Delta V\right) \cdot D \cdot \left(\frac{T_{\text{exp}}}{T_{\text{B}}}\right) [\text{J}] \tag{16}$$

6. Voltage–pressure calibration and scaling

Due to cavitation risk at experimental intensity, only low-voltage scans were performed. The pressure field is scaled from scanned conditions to experimental conditions using peak focal pressure, $P_{\text{max},\mathbf{r}_0}$:

$$E_{\text{in}}{}^{\text{exp}} = E_{\text{in}}{}^{\text{scan}} \cdot \left(\frac{P_{\text{max},\mathbf{r}_0}{}^{\text{exp}}}{P_{\text{max},\mathbf{r}_0}{}^{\text{scan}}}\right)^2 \tag{17}$$

The focal pressure at $\mathbf{r}_0$ was shown to scale linearly with voltage in the low-voltage range, as justification for this method, and is plotted in Supplementary Fig. 27.

It is noted that using sample window length τ, determined by the noise threshold, includes the ring-up and down effect of the transducer, as it resonates up to a steady state pressure amplitude during a pulse emission. This would dilute the average, affecting results if the number of sinusoid cycles, N_{cycles}, varies between the scanned data and experimental condition. Additional scaling using the scanned conditions is used to mitigate this, where $\bar{E}_{\text{PA}}(\mathbf{r})$ is substituted by $\bar{E}_{PA,\text{reconstructed}}(\mathbf{r})$ at driving frequency f_0 (Hz):

$$\bar{E}_{\text{PA,reconstructed}}(\mathbf{r}) = \tau \cdot \left(\frac{f_0}{N_{\text{cycles,scan}}}\right) \cdot \bar{E}_{\text{PA}}(\mathbf{r}) \tag{18}$$

7. Worked example

Given:

- Frequency, f_0: 820 kHz
- Peak pressure amplitude of scan, $P_{\max,\mathbf{r}_0}{}^{\text{scan}}$: 1.59 MPa
- Sample window duration, τ: 196 µs
- Number of cycles in pulse during scan, $N_{\text{cycles,scan}}$: 100 cycles
- Integrated pulse averaged density of scan, $\left(\sum_{\mathbf{r}\in V_{-6\text{dB}}} \bar{E}_{\text{PA}}(\mathbf{r}) \cdot \Delta V\right)$ = 0.4247 µJ

- Experimental peak pressure amplitude, $P_{\max,\mathbf{r}_0}{}^{\exp}$: 6.77 MPa
- Burst period, T_{B}: 2 ms
- Number of cycles in pulse during experiment, $N_{\text{cycles,scan}}$: 100 cycles
- Experimental duration, $T_{\exp}$: 30 min = 1800 s

Scale up integrated pulse averaged density using $\tau \cdot \left(\frac{f_0}{N_{\text{cycles,scan}}}\right)$:

$$0.4274 \cdot \tau \cdot \left(\frac{f_0}{N_{\text{cycles,scan}}}\right) = 0.4274 \cdot 196 \times 10^{-6} \cdot \left(\frac{820000}{100}\right) = 0.6869 \text{ µJ}$$

Apply factor $D \cdot \left(\frac{T_{\exp}}{T_{\text{B}}}\right)$ to get $E_{\text{in}}{}^{\text{scan}}$ (where $D = \frac{N_{\text{cycles,exp}}}{f_0 \cdot T_{\text{B}}}$):

$$E_{\text{in}}{}^{\text{scan}} = 0.6869 \cdot D \cdot \left(\frac{T_{\exp}}{T_{\text{B}}}\right) = 0.6869 \cdot \left(\frac{100}{820000 \times 2 \times 10^{-3}}\right) \cdot \left(\frac{1800}{2 \times 10^{-3}}\right) = 37696 \text{ µJ} = 0.0377 \text{ J}$$

Finally, multiply by pressure scaling factor:

$$E_{\text{in}}{}^{\exp} = E_{\text{in}}{}^{\text{scan}} \cdot \left(\frac{P_{\max,\mathbf{r}_0}{}^{\exp}}{P_{\max,\mathbf{r}_0}{}^{\text{scan}}}\right)^2 = 0.0377 \cdot \left(\frac{6.77}{1.59}\right)^2 = 0.68 \text{ J}$$

8. Additional acoustic intensity metrics

For comparison to other systems, characterizing the ultrasound field often involves intensity metrics, as an addition to energy-based measures. The instantaneous intensity at point **r** and time t represents the local rate of acoustic energy transport per unit area (W m^{-2}) and can be estimated from pressure as:

$$\mathbf{I}(\mathbf{r}, t) = \frac{p(\mathbf{r}, t)^{\,2}}{\rho c} \qquad (19)$$

Several useful intensity metrics are derived from this pressure-based intensity field, quantifying how this intensity varies spatially and temporally[6]:

- **Spatial-peak temporal-peak intensity (I_{SPTP})**

  The peak intensity across all space and time points:

  $$I_{\text{SPTP}} = \max_{\mathbf{r},t}\left(\frac{p(\mathbf{r}, t)^{\,2}}{\rho c}\right) \qquad (20)$$

- **Spatial-peak pulse-average intensity (I_{SPPA})**

  Time-averaged intensity at the spatial location of peak pressure during one pulse (of duration τ):

  $$I_{\text{SPPA}} = \max_{\mathbf{r}}\left(\frac{1}{\tau}\int_0^{\tau} \frac{p(\mathbf{r}, t)^{\,2}}{\rho c}\, \mathrm{d}t\right) \qquad (21)$$

- **Spatial-peak temporal-average intensity (I_{SPTA})**

At the same location as I_{SPPA}, but scaled over entire burst period:

$$I_{\mathrm{SPTA}} = \left(\frac{\tau}{T_{\mathrm{B}}}\right) \cdot I_{\mathrm{SPPA}} \tag{22}$$

- **Spatial-average pulse-average intensity ($\boldsymbol{I_{\mathrm{SAPA}}}$)**

  Time-averaged intensity averaged across the –6dB focal surface S_{6dB}, normal to the direction of wave propagation during one pulse duration:

$$I_{\mathrm{SAPA}} = \frac{1}{A_{\mathrm{6dB}}} \iint\limits_{S_{\mathrm{6dB}}} \left(\frac{1}{\tau}\int_0^{\tau} \frac{p(\mathbf{r},t)^{\,2}}{\rho c}\,\mathrm{d}t\right) ds \tag{23}$$

  where A_{6dB} is the total area of S_{6dB} (m^2).

- **Spatial-average temporal-average intensity ($\boldsymbol{I_{\mathrm{SATA}}}$)**

  Averaged over focal surface as I_{SAPA}, but scaled over entire burst period:

$$I_{\mathrm{SATA}} = \left(\frac{\tau}{T_B}\right) \cdot I_{\mathrm{SAPA}} \tag{24}$$

These intensity values for the 820 kHz and 530 kHz transducers are shown in **Supplementary Table 1.**

Supplementary Figures

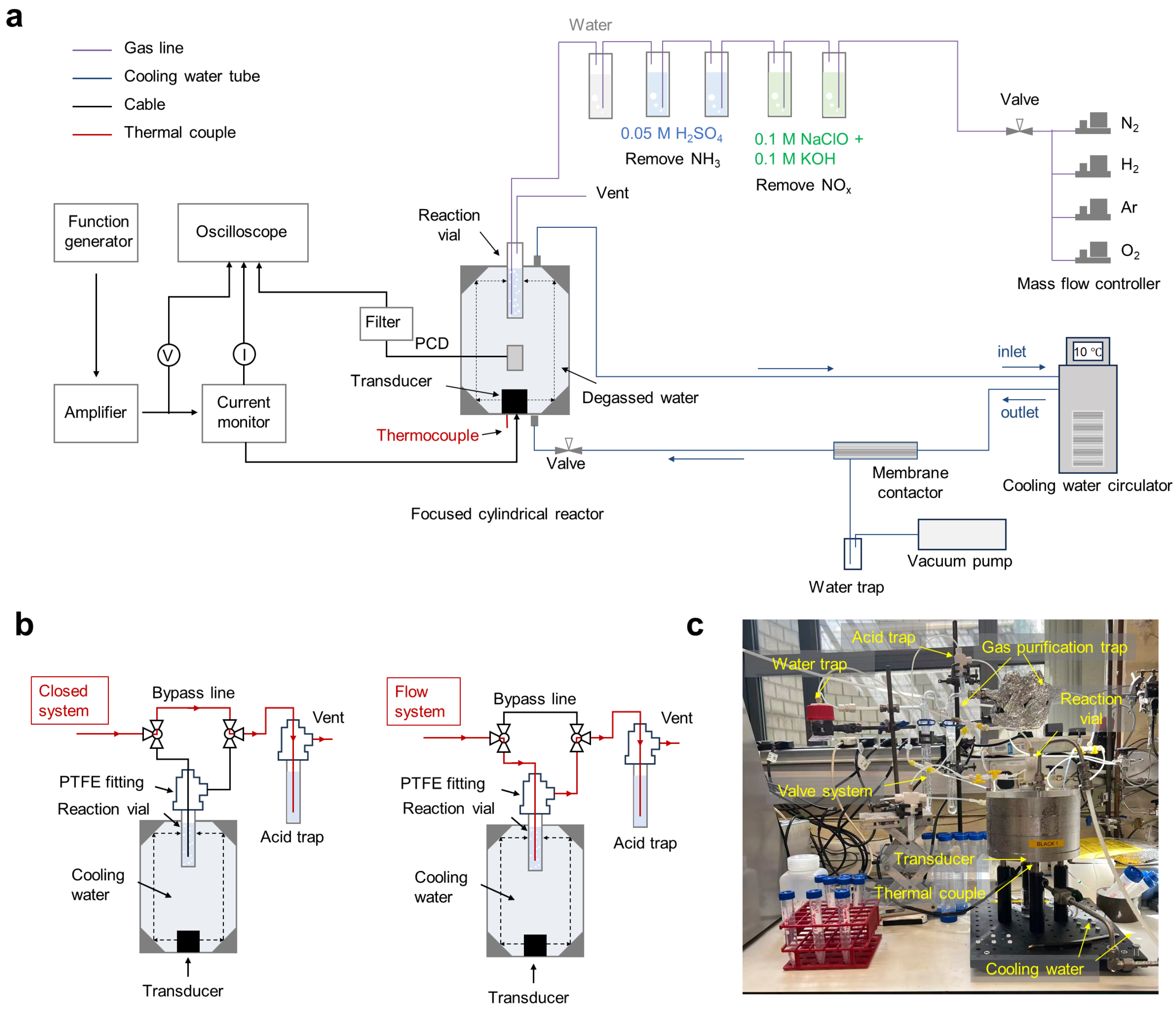

Supplementary Fig. 1. The sonochemical reactor system. a, The diagram of the sonochemical reactor system, corresponding to a continuously flow mode. **b**, The diagram of closed system and flow system. The double three-way valve system controls the flow system, that switch between continuous flow (gas passing through the reactor and into the acid trap) and closed mode (gas is first introduced to saturate the liquid, after which the reactor is isolated for ultrasound irradiation). **c**, The photograph of the sonochemical reactor.

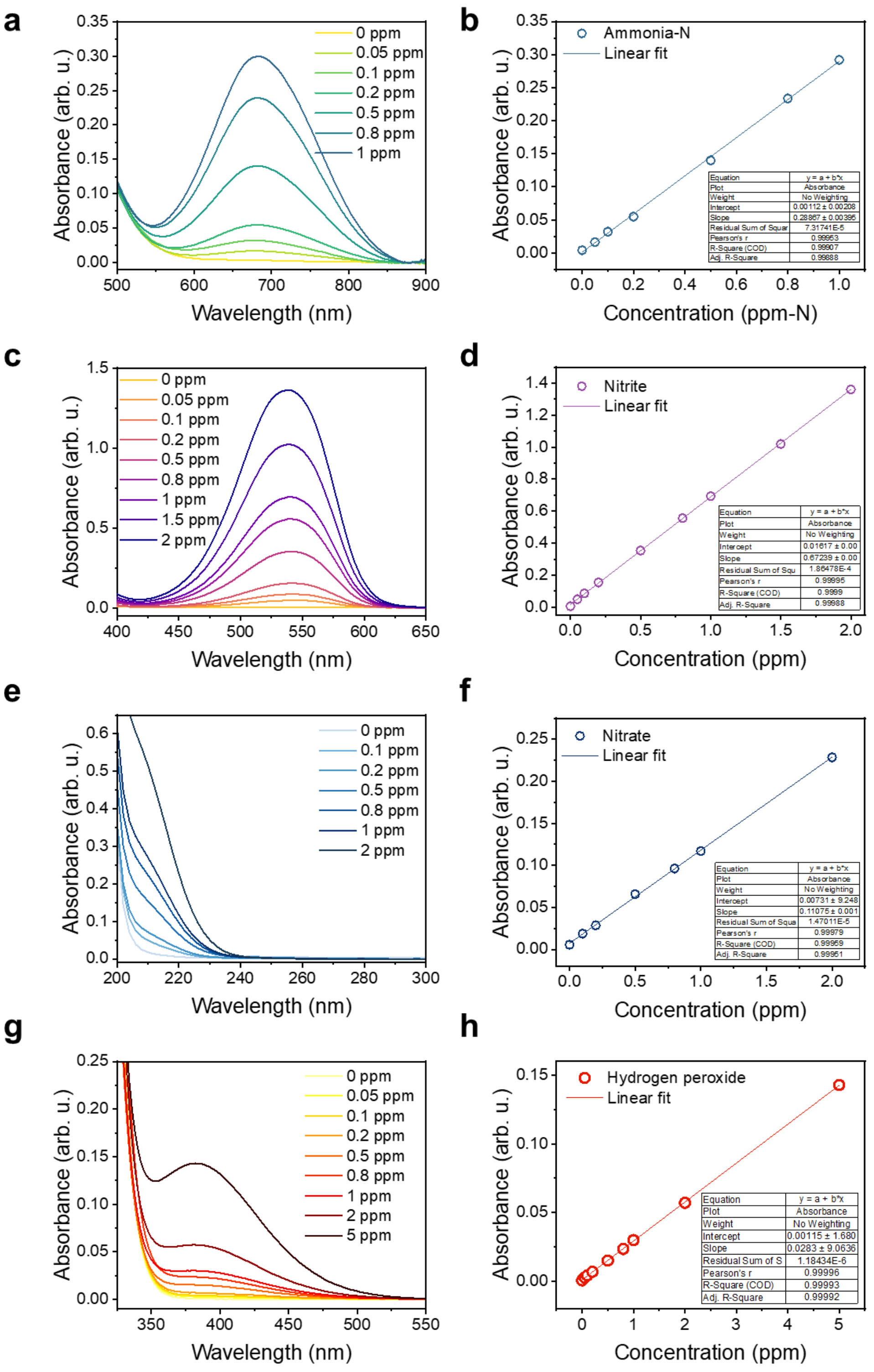

Supplementary Fig. 2. UV-vis calibration curves. a, b, UV-vis spectra and the calibration curve of ammonium solutions with varying concentrations (in ppm of N in ammonia), measured using the salicylate method. **c**, **d**, UV-vis spectra and calibration curve of potassium nitrite solutions with varying concentrations (in ppm of NO_2^-), measured using the Griess reagent. **e**, **f**, UV-vis spectra and calibration curve of potassium nitrate solutions with varying concentrations (in ppm of NO_3^-), measured after treatment with hydrochloric acid and sulfamic acid. **g**, **h**, UV-vis spectra and the calibration curve of hydrogen peroxide with varying concentrations (in ppm of H_2O_2).

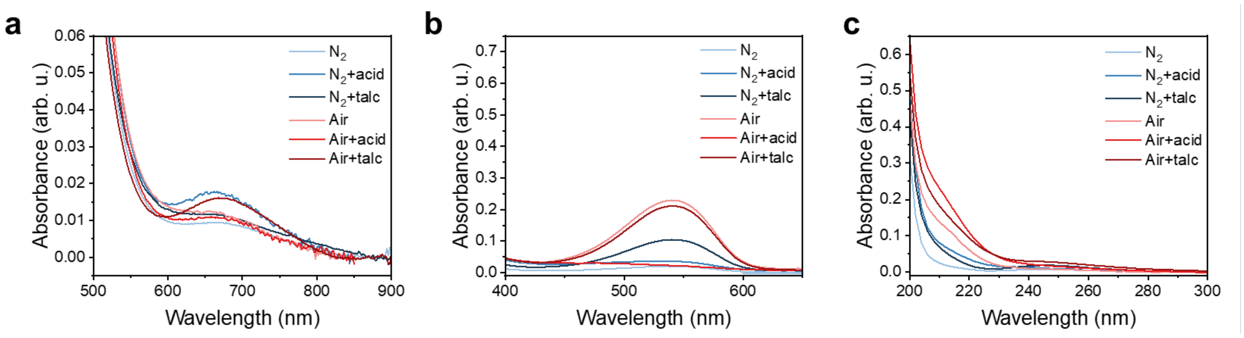

Supplementary Fig. 3. UV-vis spectra of nitrogen reaction products in different solutions. a, UV-vis spectra of ammonium produced from reactions under various conditions: "N_2" and "Air" represent reactions with pure N_2 gas and air in deionised water, respectively; "N_2 + acid" and "Air + acid" represent reactions in 0.05 M H_2SO_4 solution; "N_2 + talc" and "Air + talc" represent reactions in water containing 1 mg mL^{-1} talc. **b**, UV-vis spectra of nitrite products under the same conditions. The "Air" and "Air + talc" samples were diluted 4×, while all other samples were diluted 2× prior to measurement. **c**, UV-vis spectra of nitrate products. All samples were diluted 2× prior to measurement.

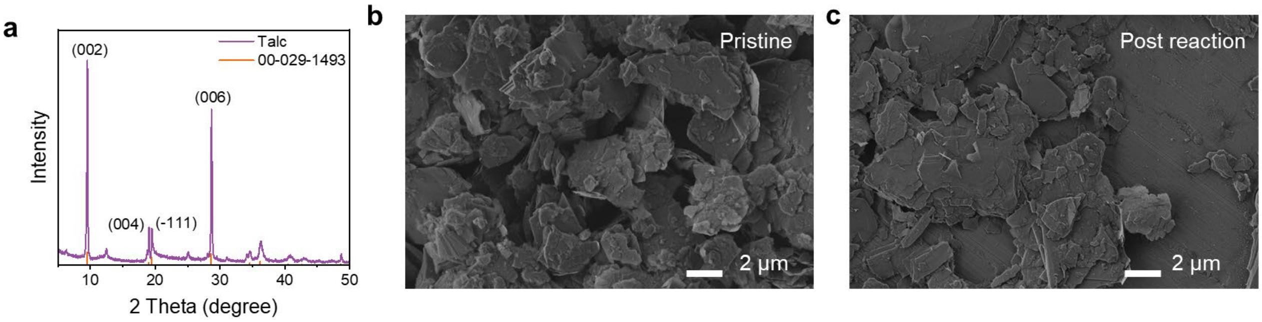

Supplementary Fig. 4. Structural characterisation of talc ($Mg_3Si_4O_{10}(OH)_2$). a, The X-ray diffraction pattern of talc, corresponding to the standard structure of PDF #00-029-1493. **b**, The SEM image of talc shows typical layered structure. **c**, SEM images of talc after 2 h sonication. The transducer was operated at 820 kHz with a 6.1% duty cycle and 6.77 MPa acoustic peak pressure.

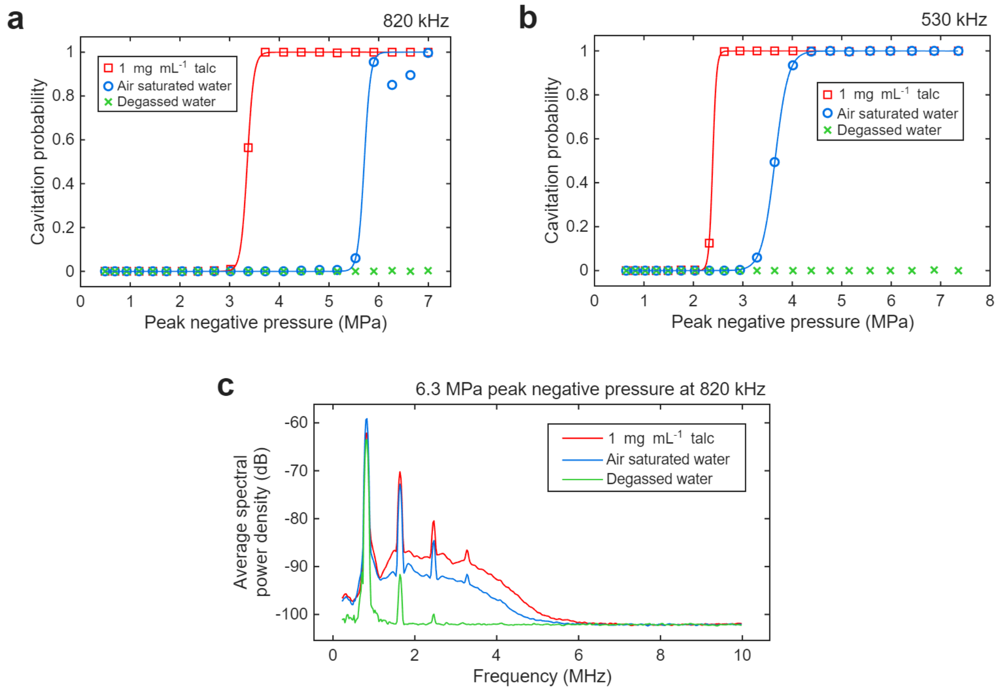

Supplementary Fig. 5. Cavitation probability as a function of peak negative acoustic pressure. Green, blue and red colours represent measurements in degassed water, air saturated water and water containing 1mg mL^{-1} talc. Symbols show measured cavitation probabilities and solid lines in figures a,b indicate sigmoidal fits to the data, using non-linear least-squares regression. **a,** Plot of driving frequencies of 820 kHz. **b**, Plot of 530 kHz. **c**, Example of power density spectra used for estimating cavitation probability, where solid lines show averaged spectra over 1000 bursts at 820 kHz and 6.3 MPa peak negative pressure.

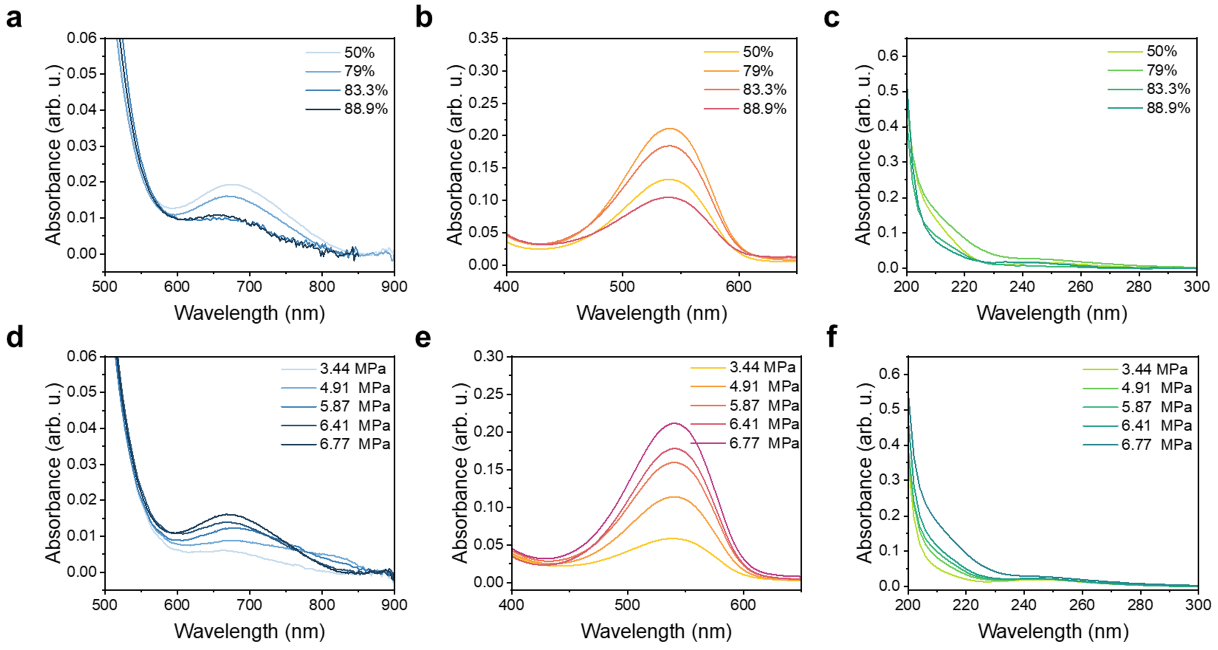

Supplementary Fig. 6. UV-vis spectra of reaction products from nitrogen–oxygen mixtures in water containing 1 mg mL^{-1} talc, under varying conditions. The nitrite samples were diluted 4×, and the nitrate samples were diluted 2× prior to measurement. **a–c,** UV-vis spectra of ammonium, nitrite, and nitrate, respectively, produced using nitrogen–oxygen mixtures with varying nitrogen fractions (indicated by percentage values). **d–f,** UV-vis spectra of ammonium, nitrite, and nitrate obtained under varying peak-to-peak acoustic pressure.

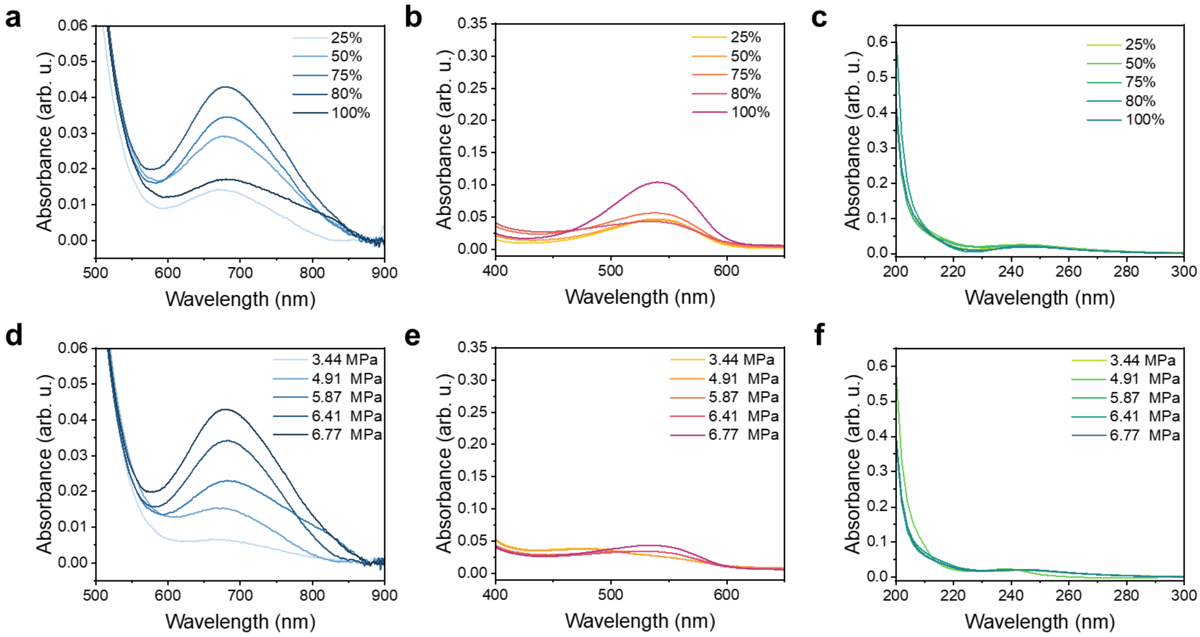

Supplementary Fig. 7. UV-vis spectra of reaction products from nitrogen–hydrogen mixtures in water containing 1 mg mL^{-1} talc, under varying conditions. Both nitrite and nitrate samples were diluted 2× prior to measurement. **a–c,** UV-vis spectra of ammonium, nitrite, and nitrate, respectively, obtained using nitrogen–hydrogen mixtures with varying nitrogen fractions (percentage values indicate nitrogen content). **d–f,** UV-vis spectra of ammonium, nitrite, and nitrate at varying peak-to-peak acoustic pressure.

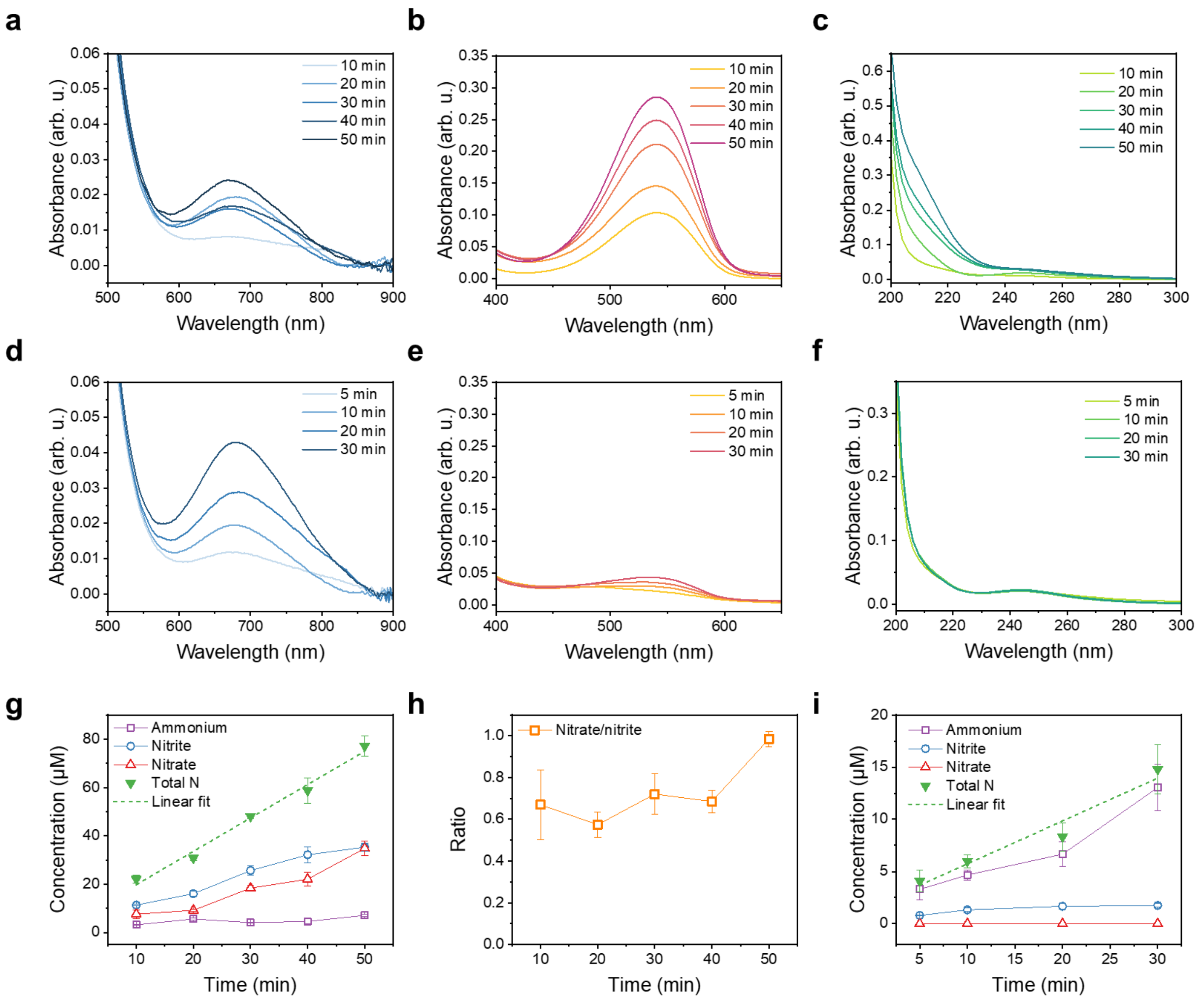

Supplementary Fig. 8. UV-vis spectra of reaction products from nitrogen–oxygen and nitrogen–hydrogen mixtures in closed reaction system. a–c, UV-vis spectra of ammonium, nitrite, and nitrate from nitrogen–oxygen feed gas with different reaction durations. The ammonium samples were not diluted, except 10-min sample were diluted 2×, nitrite samples were diluted 4×, and the nitrate samples were diluted 2× prior to measurement. **d–f,** UV-vis spectra of ammonium, nitrite, and nitrate from nitrogen–hydrogen feed gas with different reaction durations. Both nitrite and nitrate samples were diluted 2× prior to measurement. **g,** Product concentrations as a function of reaction time using air as the feed gas. **h,** The corresponding nitrate-to-nitrite ratio against time. **i,** Product concentrations as a function of reaction time with nitrogen–hydrogen as the feed gas. Error bars represent the standard deviation from three independent measurements, applied consistently across all subsequent scatter plots. The individual data points can be found in the Source Data file.

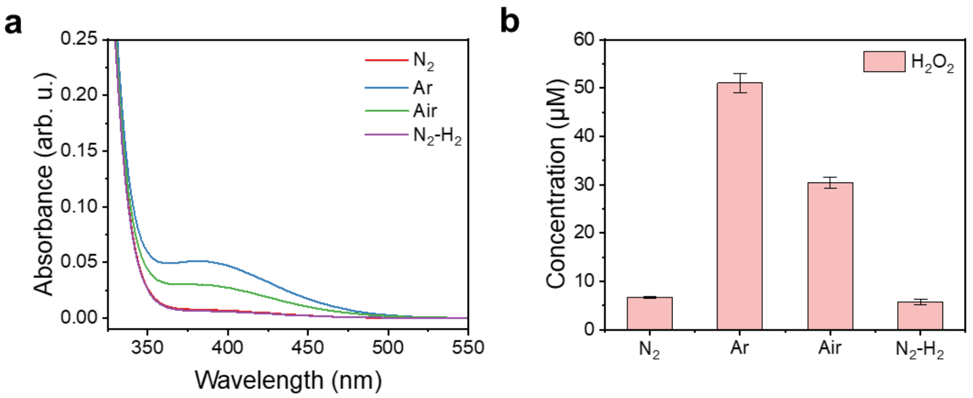

Supplementary Fig. 9. Hydroperoxide production from different feed gases. a, UV-vis spectra of the hydrogen peroxide with different feed gases after 30 min reaction. **b,** The calculated concentration of hydrogen peroxide. Error bars represent the standard deviation from three independent measurements. The individual data points can be found in the Source Data file.

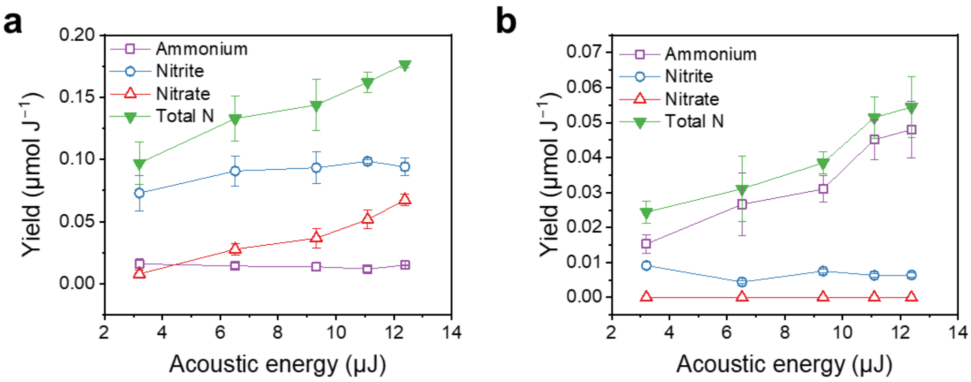

Supplementary Fig. 10. The plot of nitrogen fixation yield per unit energy against pulse energy. The pulse energy represents the acoustic energy delivered into the reaction vial during each 100-cycle burst, calculated based on the 3D acoustic pressure field scan (see Supplementary note 2 for details). **a,** The production in closed reaction system with air as feed gas. **b,** The production in closed reaction system with nitrogen-hydrogen mixtures (80% nitrogen) as feed gas. Error bars represent the standard deviation from three independent measurements. The individual data points can be found in the Source Data file.

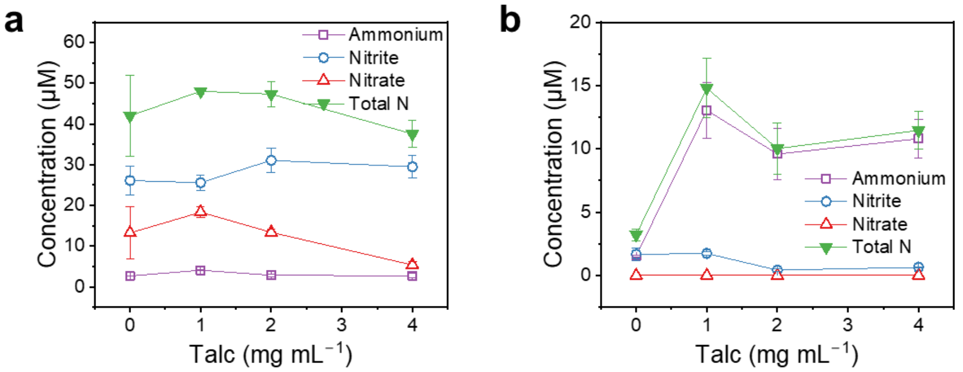

Supplementary Fig. 11. The plot of nitrogen fixation against talc concentration. a, The production in closed reaction system with air as feed gas in water with different talc concentration. **b,** The production in closed reaction system with nitrogen–hydrogen mixtures (80% nitrogen) as feed gas in water with different talc concentration. Error bars represent the standard deviation from three independent measurements. The individual data points can be found in the Source Data file.

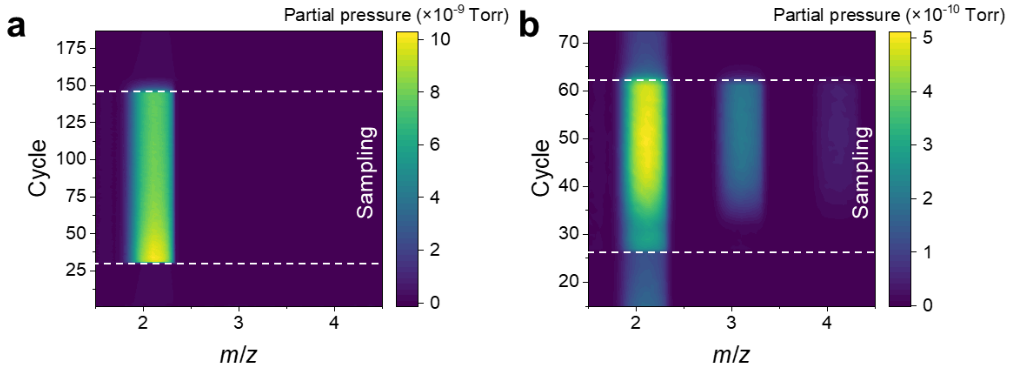

Supplementary Fig. 12. Contour plots of mass spectra of products with argon as the feed gas. a, Hydrogen production in H_2O, **b,** Products in mixed D_2O+H_2O solution.

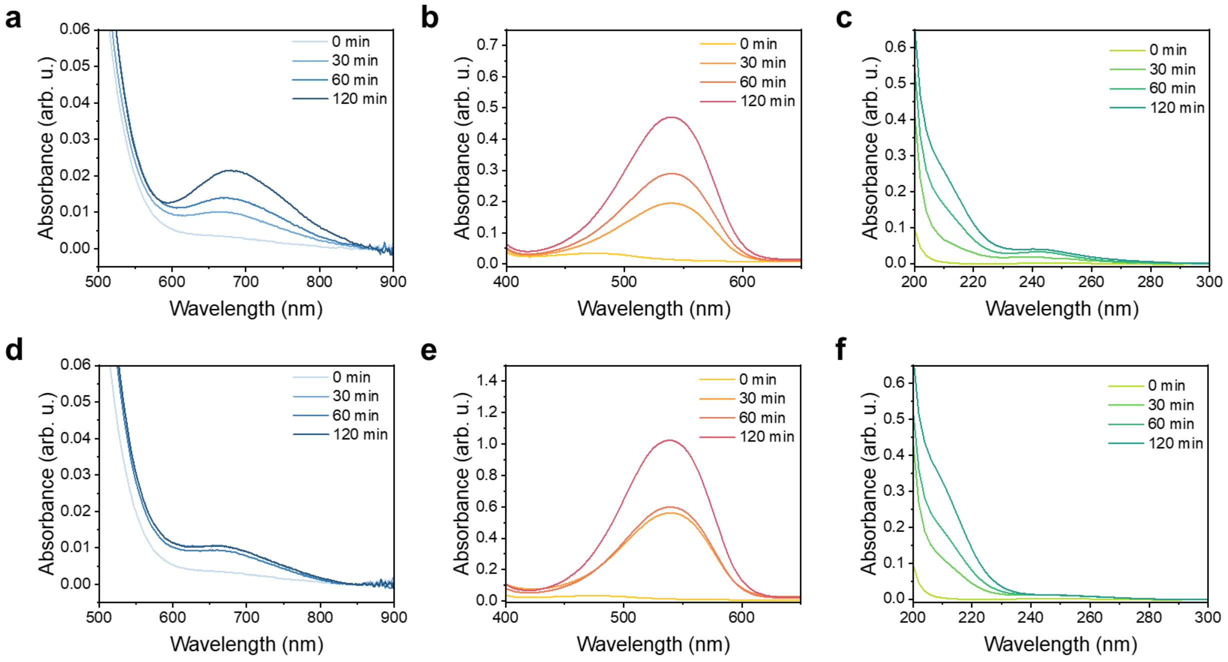

Supplementary Fig. 13. UV-vis spectra of reaction products from pure nitrogen and nitrogen–oxygen mixtures in water containing 1 mg mL^{-1} talc. a–c, UV-vis spectra of ammonium, nitrite, and nitrate produced from pure nitrogen at varying reaction times. **d–f,** UV-vis spectra of ammonium, nitrite, and nitrate from a nitrogen–oxygen mixture (79% nitrogen) at varying reaction times. Nitrite and nitrate solutions from the 30-minute reactions were diluted 2×, while those from the 60- and 120-minute reactions were diluted 4× prior to measurement.

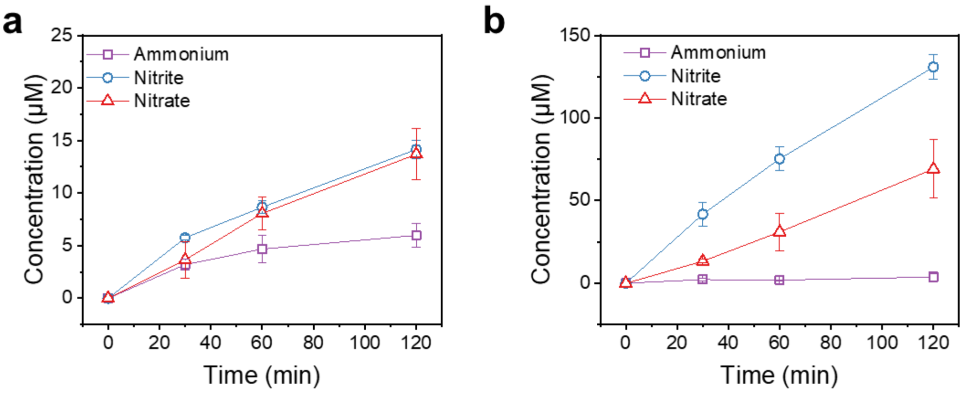

Supplementary Fig. 14. The plot of products against time in continuous gas flow reaction system. a, Pure nitrogen as feed gas, **b,** air as feed gas. Error bars represent the standard deviation from three independent measurements. The individual data points can be found in the Source Data file.

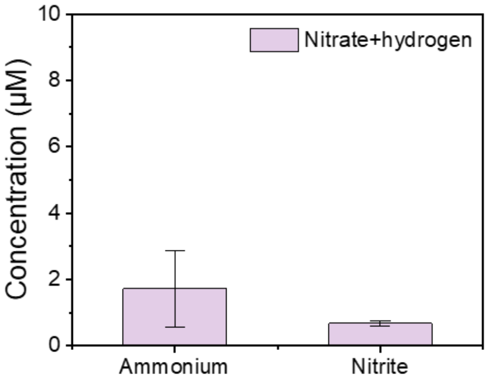

Supplementary Fig. 15. The plot of products from nitrate reduction with H_2 as feed gas. Nitrate solution is 10 mM and reaction is performed in continuous gas flow reaction system. Error bars represent the standard deviation from three independent measurements. The individual data points can be found in the Source Data file.

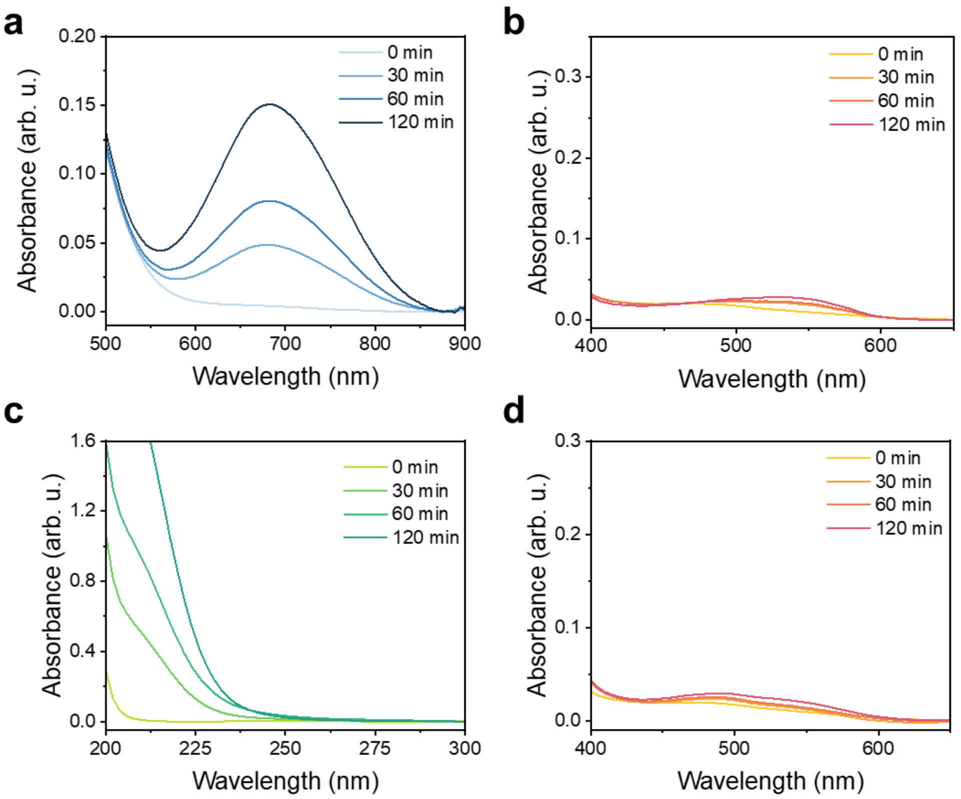

Supplementary Fig. 16. UV-vis spectra of reaction products under continuous gas flow. a, **b**, UV-vis spectra of products from nitrogen–hydrogen mixtures (80% nitrogen) in neutral water containing 1 mg mL^{-1} talc. Nitrite solutions were diluted 2× prior to measurement. **c**, **d**, UV-vis spectra of nitrate and nitrite from reaction in air in acidic water (0.05 M H_2SO_4) containing 1 mg mL^{-1} talc.

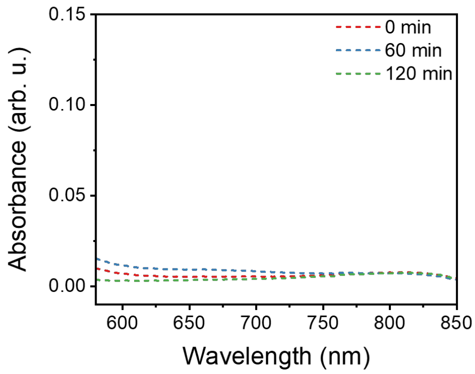

Supplementary Fig. 17. UV–vis absorbance curves from ammonium assays of the acid trap (dashed line), showing detectable ammonium only in the reaction solution. Conditions: feed gas is 80% N_2 and 20% H_2, frequency is 820 kHz, and duty cycle is 6.1%.

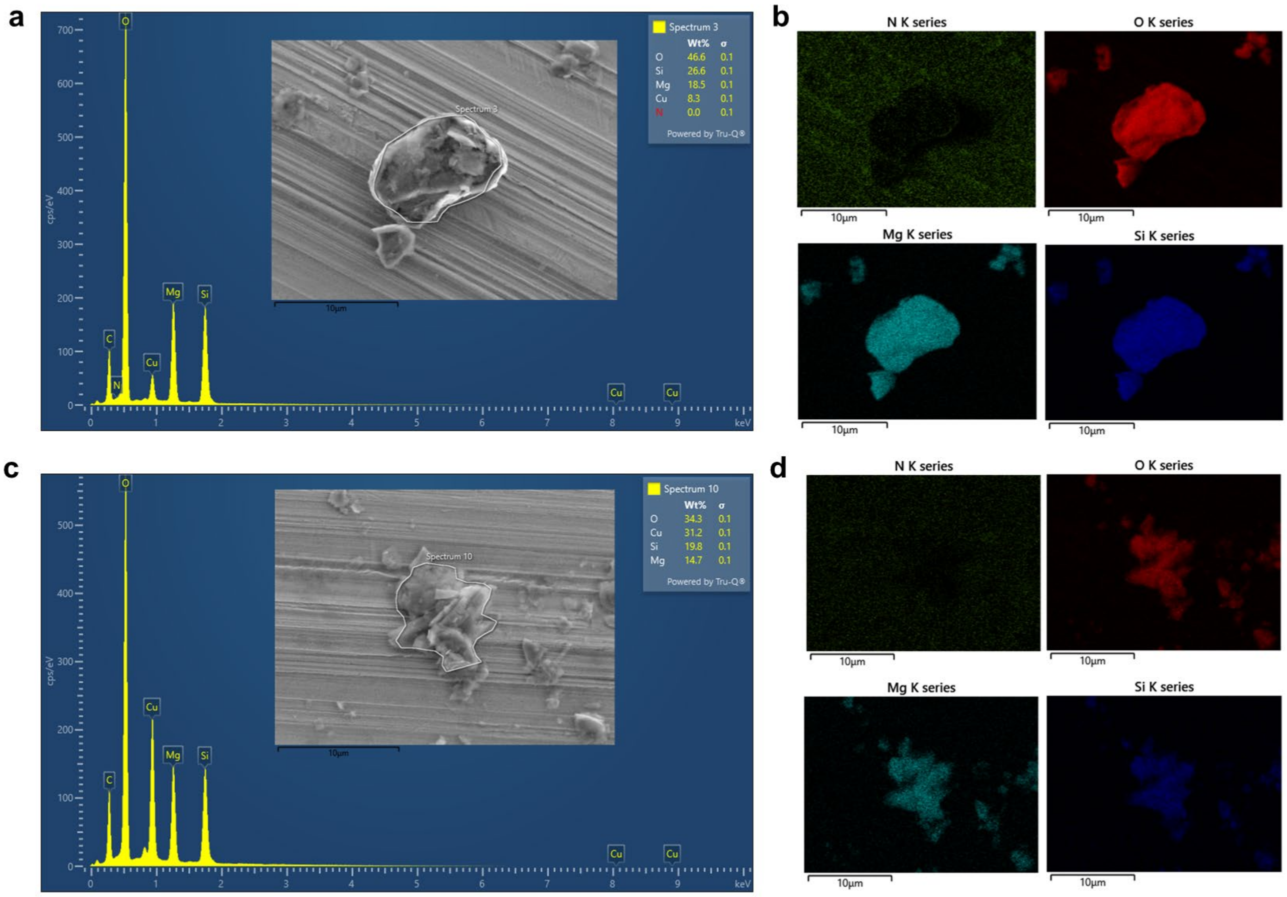

Supplementary Fig. 18. Energy-dispersive X-ray spectroscopy (EDX) mapping plots. a, b, Pristine talc figures and **c-d,** after 2 h sonication. For the pristine talc, element atomic ratios are N 0%, O 61.32%, Mg 16.05%, Si 19.89%, Cu (Substrate) 2.74%. For the talc after 2 h sonication, N 0%, O 54.37%, Mg 15.35%, Si 17.83%, Cu (Substrate) 12.45%.

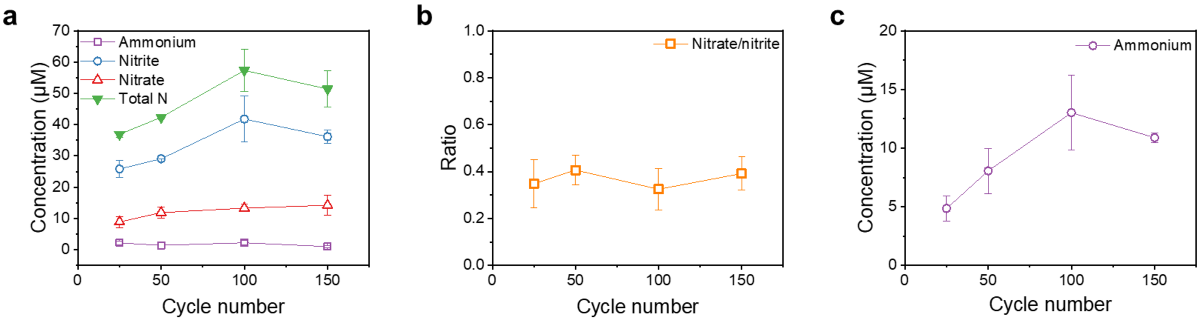

Supplementary Fig. 19. Products concentration from reaction with different pulse length. All reactions were carried out for 30 minutes at 6.77 MPa acoustic peak pressure, duty cycle 6.1% in neutral water with 1 mg mL^{-1} talc. **a,** N_2–O_2 mixtures in neutral water different pulse length with the same duty cycle. **b,** The corresponding nitrate-to-nitrite ratio. **c,** N_2–H_2 (80% nitrogen) mixtures in neutral water with different pulse length with the same duty cycle. Error bars represent the standard deviation from three independent measurements. The individual data points can be found in the Source Data file.

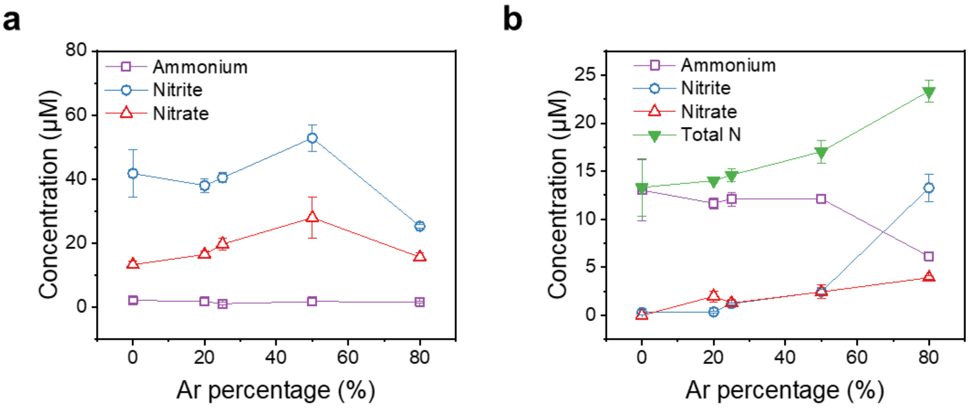

Supplementary Fig. 20. Products from reactions with varying argon percentages in gas mixtures. All reactions were carried out for 30 minutes at 6.77 MPa acoustic peak pressure, duty cycle 6.1% in neutral water with 1 mg mL^{-1} talc. **a,** Nitrogen products from reactions using air–Ar mixtures with varying Ar fractions. **b,** Nitrogen products from N_2–H_2–Ar mixtures (80% N_2–20% H_2 base mixture with varying Ar content). Error bars represent the standard deviation from three independent measurements. The individual data points can be found in the Source Data file.

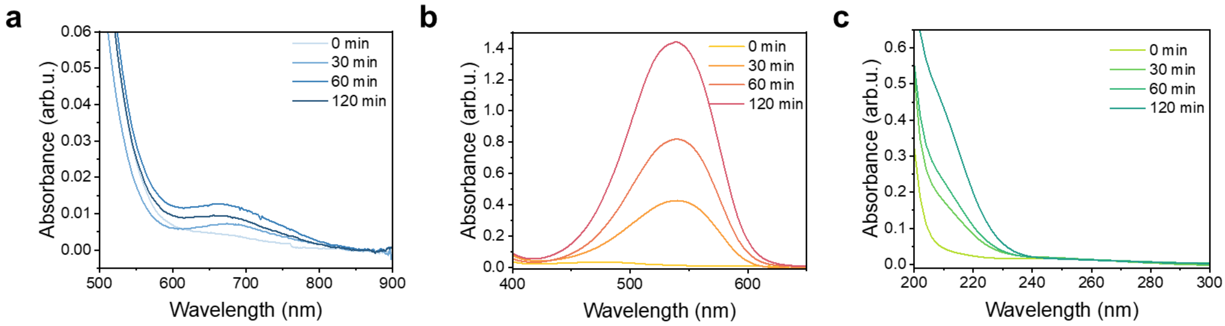

Supplementary Fig. 21. UV-vis spectra of reaction products from air–argon mixtures in water containing 1 mg mL^{-1} talc. All reactions were carried out at 6.77 MPa acoustic peak pressure, duty cycle 6.1% for different duration in neutral water with 1 mg mL^{-1} talc. **a–c,** UV-vis spectra of ammonium, nitrite, and nitrate produced from 50% N_2–50% Ar at varying reaction times. Ammonium solutions were not diluted, while nitrite and nitrate samples were diluted 2× prior to measurement.

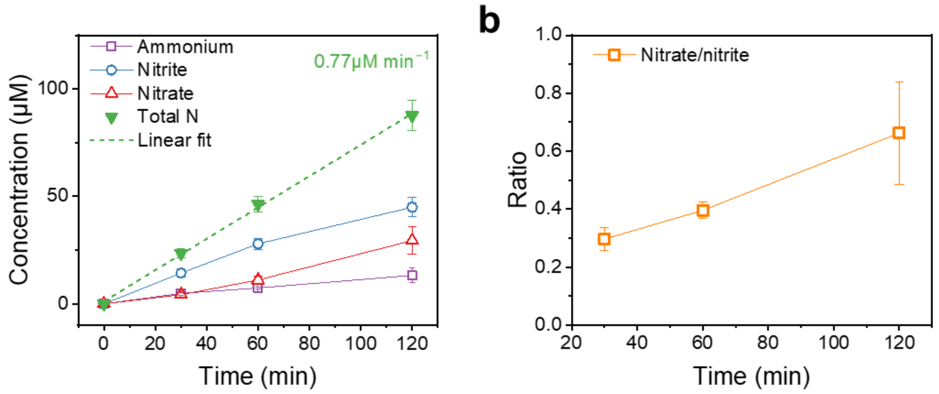

Supplementary Fig. 22. The nitrogen fixation with 50% N_2 and 50% Ar as feed gas. All reactions were carried out at 6.77 MPa acoustic peak pressure, duty cycle 6.1% for different duration in neutral water with 1 mg mL^{-1} talc. **a,** Total fixed nitrogen and **b,** nitrate-to-nitrite ratios from reactions. Error bars represent the standard deviation from three independent measurements. The individual data points can be found in the Source Data file.

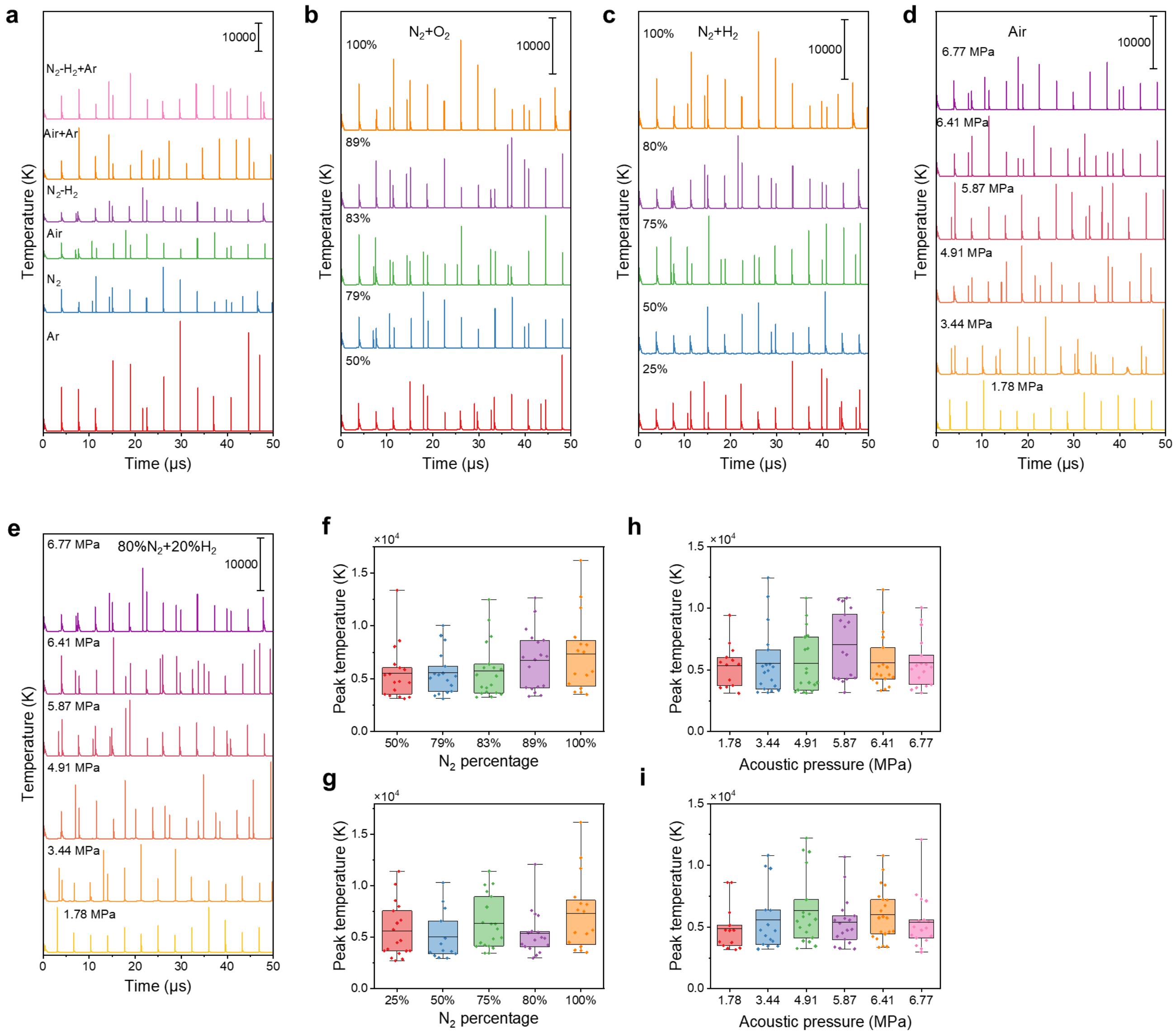

Supplementary Fig. 23. Simulated temperature profiles during cavitation bubble collapse under various conditions. a, Temperature profiles for different gas mixtures during a single bubble collapse event. **b,** Temperature profiles for N_2–O_2 mixtures with varying nitrogen content (indicated percentages refer to N_2 fraction). **c,** Temperature profiles for N_2–H_2 mixtures with varying nitrogen content. **d,** Temperature profiles for air as the feed gas under different acoustic pressure, corresponding to experimental conditions. **e,** Temperature profiles for N_2–H_2 as the feed gas under different acoustic pressure, corresponding to experimental conditions. **f-i,** Corresponding peak temperatures extracted from the simulations in figures **b**–**e**, illustrating the influence of gas composition and ultrasound power on maximum bubble temperature. Boxes indicate the interquartile range, the central line denotes the mean value, and whiskers represent the full minimum–maximum span.

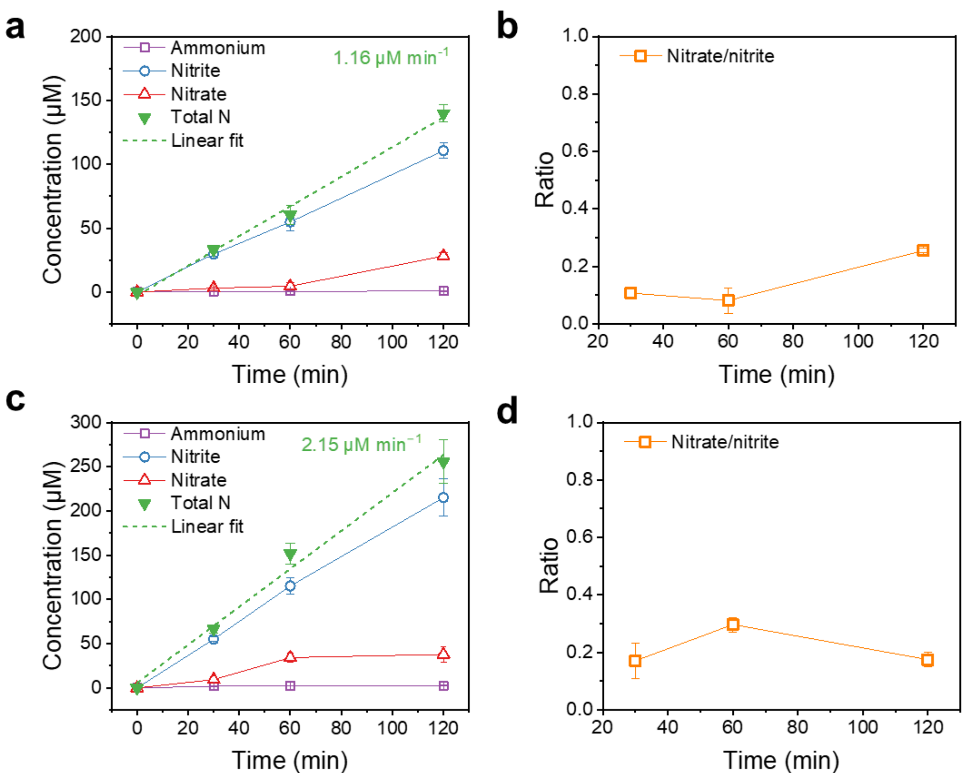

Supplementary Fig. 24. Comparison of nitrogen fixation products with and without argon obtained with 530 kHz transducer. Experiments using the 530 kHz transducer were conducted with a burst period of 3 ms and a duty cycle of 6.3%, the maximum acoustic pressure is 6.24 MPa. **a,** Product distribution using air as the feed gas, the R^2 value for linear fitting is 0.994. **b,** The corresponding nitrate-to-nitrite ratio. **c,** Product distribution using 50% air and 50% Ar gas mixture as the feed gas, the R^2 value for linear fitting is 0.989. **d,** The corresponding nitrate-to-nitrite ratio. Error bars represent the standard deviation from three independent measurements. The individual data points can be found in the Source Data file.

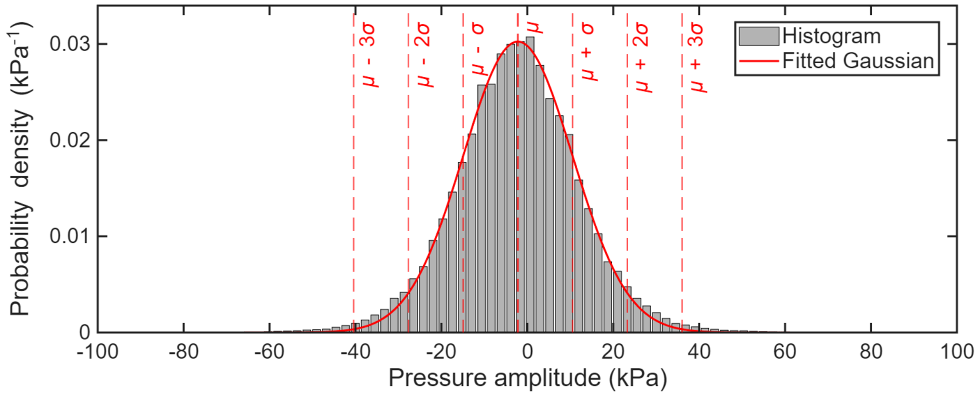

Supplementary Fig. 25. Histogram of baseline noise pressure amplitudes (grey) with Gaussian fit in red solid line. Vertical dashed lines show μ, $\mu \pm \sigma$, $\mu \pm 2\sigma$, and $\mu \pm 3\sigma$ pressure values.

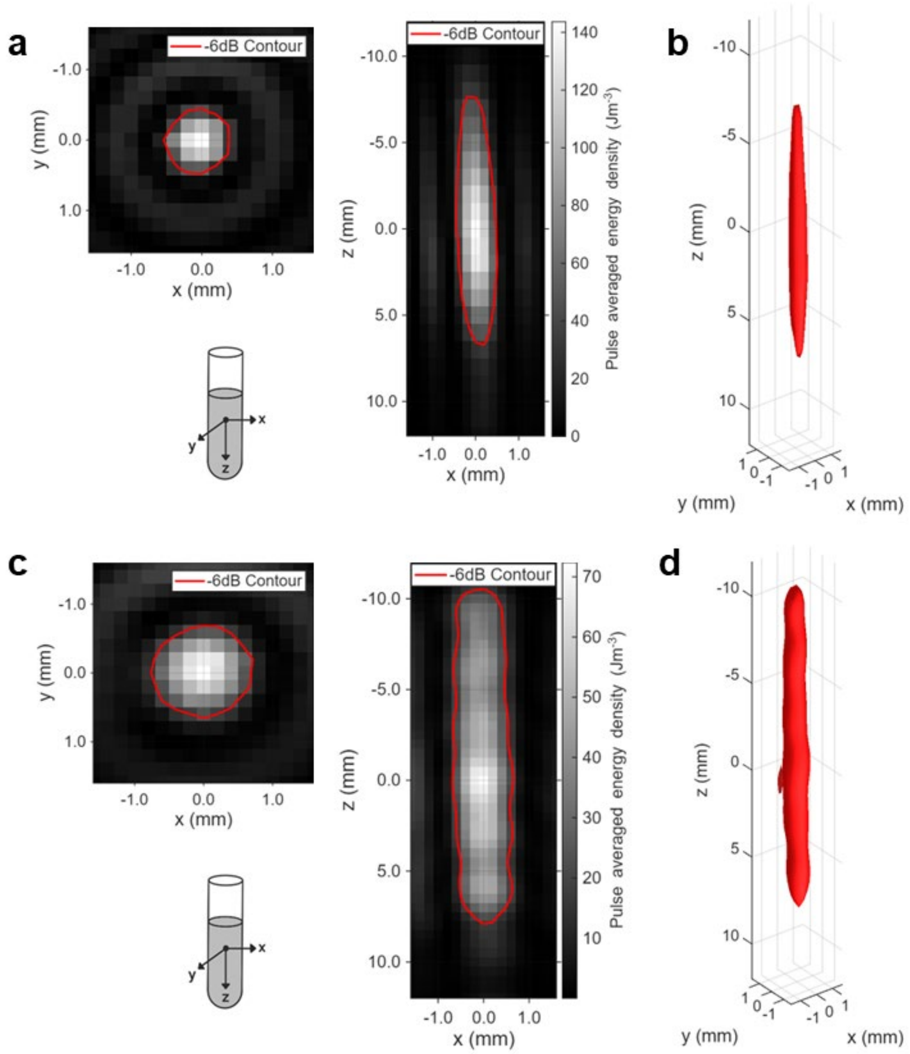

Supplementary Fig. 26. 3D scan of the energy density distribution in the reaction vial. a, **c,** pulse averaged energy density (J m^{-3}) distribution across 2D planes passing through point $\mathbf{r}_0 = (0,0,0)$ where energy density is spatially maximum. Red contour defines 2D boundary, where local energy density has fallen by 6 dB relative to spatial maximum. **b**, **d** show the 3D volume of the same boundary. **a**, **b,** Data of 820 kHz transducer, with 1.59 MPa peak pressure amplitude at $\mathbf{r}_0$. **c**, **d,** Data of 530 kHz transducer, with 1.33 MPa peak pressure amplitude at $\mathbf{r}_0$. NB: maximum energy density at 530 kHz is approximately half of 820 kHz, as voxel size used for analysis was half the volume. The −6dB volume for 820 kHz and 530 kHz is 6.16 mm^3 and 19.74 mm^3, respectively.

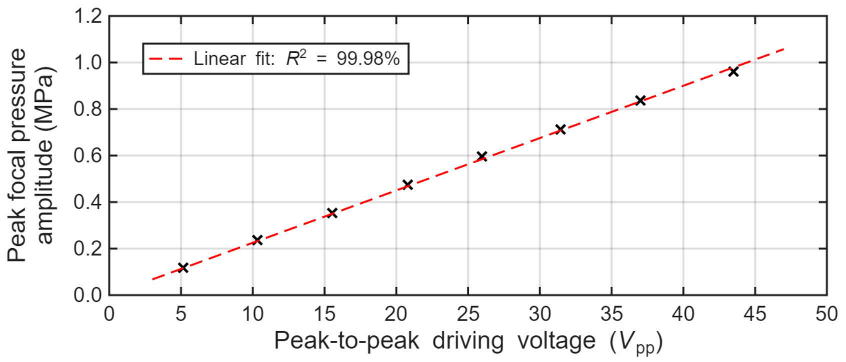

Supplementary Fig. 27. Plot of focal pressure vs. input voltage, at 820 kHz transducer driving frequency. Linear relationship is shown, as justification for pressure scaling for higher experimental voltages (R^2=0.998).

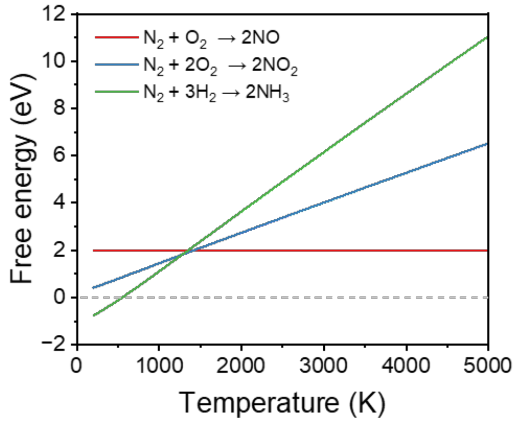

Supplementary Fig. 28. Gibbs free energy changes (Δ*G*) for the formation of NO, NO_2, and NH_3 as a function of temperature. These results highlight the limited favourability of product formation at constant high temperatures.

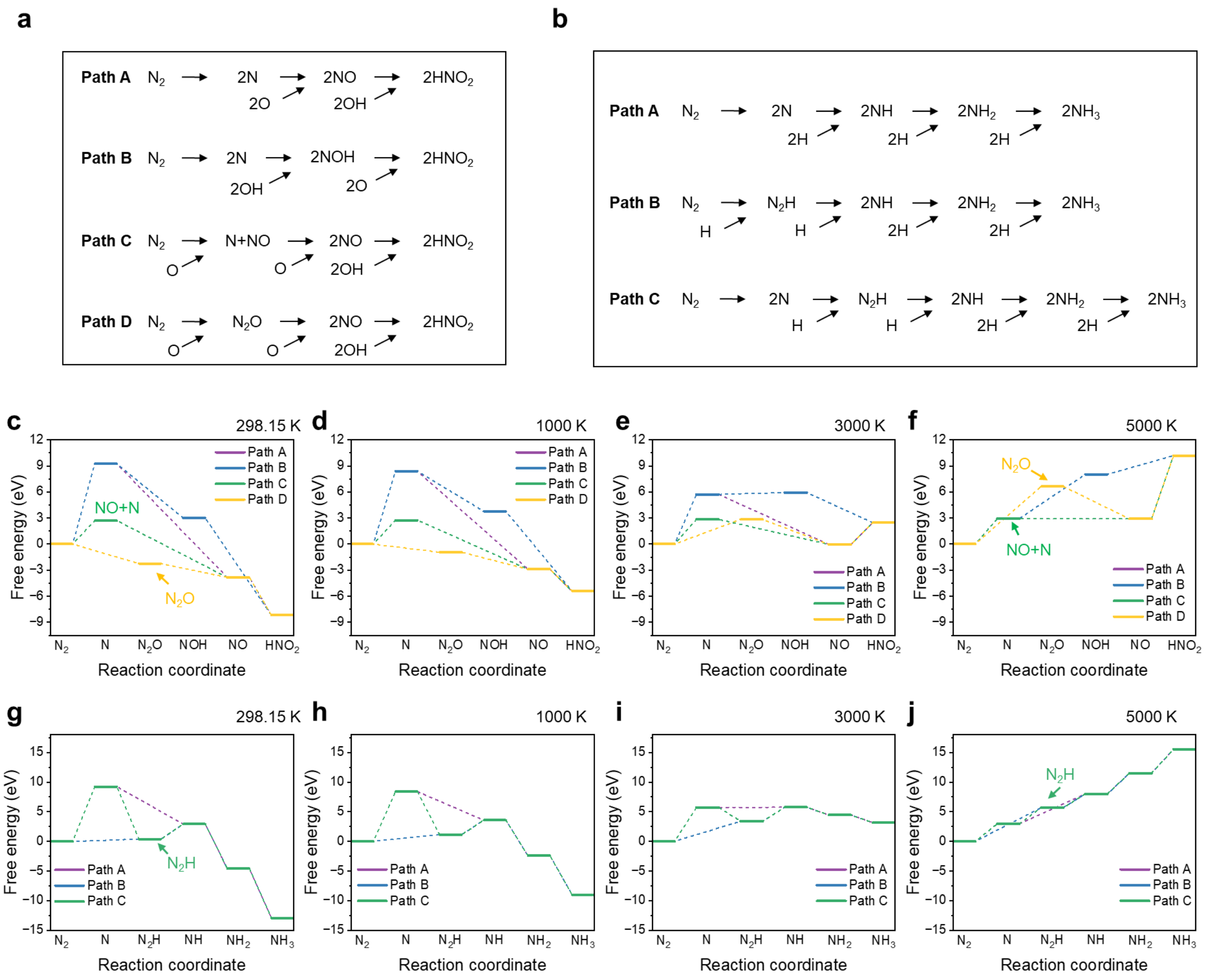

Supplementary Fig. 29. Gibbs free energy profiles for nitrous acid and ammonia production via different reaction pathways. a, The proposed different reaction pathways for nitrous acid production. **b,** The proposed different reaction pathways for ammonia production. **c–f,** Free energy steps of representative nitrogen oxidation pathways leading to the formation of HNO_2 under varying mechanistic routes. **g–j,** Free energy steps of nitrogen reduction pathways leading to the formation of NH_3. These calculations illustrate the thermodynamic feasibility of each step, providing insight into how pathway accessibility shifts with reaction conditions.

Supplementary Tables

Supplementary Table 1. **Computed intensity metrics from simulated acoustic field.** 820kHz values based on a 6.1% duty cycle, and 530 kHz a 6.3% duty cycle. Time-averaged values are dependent on pulsing protocol and should be interpreted accordingly.

Frequency (kHz)	$P_{\max,\mathbf{r0}}$ (MPa)	I_{SPTP} (W cm^{-2})	I_{SPPA} (W cm^{-2})	I_{SPTA} (W cm^{-2})	I_{SAPA} (W cm^{-2})	I_{SATA} (W cm^{-2})	A_{6dB} (cm^2)	V_{6dB} (mm^3)
820	1.59	169.47	43.11	4.22	17.31	1.06	0.33	6.16
530	1.33	118.49	21.71	2.84	11.54	0.73	0.66	19.74

Supplementary Table 2. The summary of N_2 fixation rate with ultrasound technology

Products	Frequency (kHz)	Power (W)	Gas	Liquid	Production rate (μM min^{-1})	Power-normalised production rate (μM min^{-1} W^{-1})	Energy cost (MJ mol^{-1})	Energy-normalised production (mol kWh^{-1})	Reference
Total N	820	12.92*	50%Air, 50%Ar	Water	39.67	0.19	3.75	0.96	This work
Total N	820	12.92*	Air	Water	27.70	0.13	5.36	0.67	This work
Nitrate	820	12.92*	Air	Water + 0.05 M H_2SO_4	16.89	0.080	8.80	0.41	This work
Ammonium	820	12.92*	80%N_2, 20%H_2	Water	7.38	0.035	20.14	0.18	This work
Total N	530	12.94*	50%Air, 50%Ar	Water	34.13	0.16	9.21	0.39	This work
Total N	530	12.94*	Air	Water	18.41	0.087	17.07	0.21	This work
Ammonia	1700	18	N_2	Water	0.13	7.01×10^{-3}	1.22×10^{5}	2.94×10^{-5}	7
Nitrite	1700	18	N_2	Water	0.04	1.98×10^{-3}	4.32×10^{5}	8.33×10^{-6}	7
Nitrate	1700	18	N_2	Water	0.07	4.10×10^{-3}	2.09×10^{5}	1.72×10^{-5}	7
Ammonia	1700	18	Air	Water	0.12	6.48×10^{-3}	1.32×10^{5}	2.72×10^{-5}	7
Nitrite	1700	18	Air	Water	0.64	0.036	2.40×10^{4}	1.50×10^{-4}	7
Nitrate	1700	18	Air	Water	0.86	0.048	1.80×10^{4}	2.00×10^{-4}	7
Ammonia	850	80	60%N_2, 40%H_2	Water	0.51	6.32×10^{-3}	2.50×10^{4}	1.44×10^{-4}	8
Ammonia	850	80	60%N_2, 40%H_2	Mineral oil	0.02	2.47×10^{-4}	6.38×10^{5}	5.64×10^{-6}	8
Nitrite	35	-	Air	Water	0.38	-	-	-	9
Nitrate	35	-	Air	Water	0.15	-	-	-	9
Nitrite	447	50	Air	Water	22	0.44	-	-	10
Nitrate	447	50	Air	Water	6	0.12	-	-	10
Nitrate	40	240	N_2	Water + 50 μM $FeSO_4$	0.21	8.75×10^{-4}	-	-	11
Nitrate	40	240	Air	Water + 50 μM $FeSO_4$	0.12	5.00×10^{-4}	-	-	11
Nitrite	360	50	36%N_2, 64%Ar	Water	1.56	0.031	7.69×10^{3}	4.68×10^{-4}	12
Nitrite	360	50	54%N_2, 46%Ar	Water	2.04	0.041	5.88×10^{3}	6.12×10^{-4}	12
NO_x	900	27	60%N_2, 40%O_2	Water	8.00	0.296	2.00×10^{3}	1.80×10^{-3}	13
NO_x	200	149 W L^{-1}	Air	Water	21	-	426	8.45×10^{-3}	14
NO_x	40	33.5 W L^{-1}	Air	Water	4.5	-	449	8.02×10^{-3}	14

*The presented power is nominal power, defined in Supplementary Note 1.

Supplementary Table 3. Comparison between nitrogen-fixation approaches (In addition to Haber-Bosch, other methods are aqueous solutions based)

Process	Reaction	Typical conditions	Selectivity	Yields	Reference
Haber–Bosch (industrial)	Reduction to NH_3	400–500 °C, 150–300 atm, Fe catalysts, H_2 from fossil sources	≈100% selectivity (NH_3), 15% per-pass conversion	Tonne-scale continuous production	15,16
Electrocatalytic N_2 reduction (aqueous)	Reduction to NH_3	Ambient condition, transition metal catalysts	1–20% selectivity (estimated from faradic efficiency)	10^2–10^5 µmol h^{-1} g_{cat}^{-1}	17-19
Photocatalytic N_2 reduction (aqueous)	Reduction to NH_3	Ambient condition, semiconductor photocatalysts + light	1–10% (dominated by H_2 production)	1–10^3 µmol h^{-1} g_{cat}^{-1}	20
Plasma reduction (gas-phase or plasma-liquid hybrid)	Reduction to NH_3	Ambient condition, non-thermal plasma activating N_2 + hydrogen source (H_2 or H_2O)	<20% (mixed with NO_x H_2, O_3 and H_2O_2)	10^2–10^3 µmol h^{-1} g_{cat}^{-1}	21,22
Mechanochemical N_2 reduction	Reduction to NH_3	Ball milling with catalysts under N_2 and H_2/H_2O	Up to 99% (rarely reported)	≈250 µmol h^{-1} g_{cat}^{-1}	23,24
Sonochemical reaction (N_2-H_2 mixtures, aqueous)	Reduction to NH_3	Ambient conditions, high-intensity ultrasound	<80% (estimated from detectable products)	1.11 µmol h^{-1}, 0.18 mol kWh^{-1}	This work
Birkeland–Eyde	Oxidation to NO_x and conversion to HNO_3	Electric arc at >3000 K, rapid quenching, atmospheric air	No specific data (newly reported data 84% NO_2)	≈60 MWh per tonne HNO_3	25,26
Electrochemical oxidation of N_2 (aqueous)	Oxidation to nitrite/nitrate in solution	Ambient conditions, catalyst/radical pathways	<30% selectivity (estimated from faradic efficiency)	10–10^3 µmol h^{-1} g_{cat}^{-1}	27,28
Plasma oxidation (gas-phase or plasma-liquid hybrid)	Oxidation to nitrate/nitrite in solution	Ambient conditions, plasma discharge in air or on water interface	20–99% (mixture of NO_2^-, NO_3^-, H_2O_2)	10^{-1}–10^5 mg_{NOx} h^{-1}	29-31
Sonochemical oxidation system (air/air–Ar mixtures)	Oxidation to nitrate/nitrite in solution	Ambient conditions, high-intensity ultrasound	40-70% (mixture of NO_2^-, NO_3^-, H_2O_2)	27.7 µmol h^{-1}, 0.67 mol kWh^{-1}	This work

Supplementary References

1. Haller, J., Wilkens, V. & Shaw, A. Determination of acoustic cavitation probabilities and thresholds using a single focusing transducer to induce and detect acoustic cavitation events: I. Method and terminology. *Ultrasound. Med. Biol.* **44**, 377–396 (2018).
2. Martínez, R. F., Cravotto, G. & Cintas, P. Organic sonochemistry: a chemist's timely perspective on mechanisms and reactivity. *J. Org. Chem. Res.* **86**, 13833–13856 (2021).
3. Schenke, S., Melissaris, T. & Van Terwisga, T. On the relevance of kinematics for cavitation implosion loads. *Phys. Fluids* **31**, (2019).
4. Kerboua, K., Hamdaoui, O. & Alghyamah, A. Energy balance of high-energy stable acoustic cavitation within dual-frequency sonochemical reactor. *Ultrason. Sonochem.* **73**, 105471 (2021).
5. Acevedo, P. & Das-Gupta, D. The measurement of the spatial average temporal average intensity Isata and ultrasonic power W in composite ultrasonic transducers for medical application. *Ultrason.* **40**, 819–821 (2002).
6. Harris, G. R. A discussion of procedures for ultrasonic intensity and power calculations from miniature hydrophone measurements. *Ultrasound. Med. Biol.* **11**, 803–817 (1985).
7. Wang, Y. *et al.* Catalyst-free nitrogen fixation by microdroplets through a radical-mediated disproportionation mechanism under ambient conditions. *J. Am. Chem. Soc.* **147**, 2756–2765 (2025).
8. Supeno & Kruus, P. Fixation of nitrogen with cavitation. *Ultrason. Sonochem.* **9**, 53–59 (2002).
9. Wakeford, C. A., Blackburn, R. & Lickiss, P. D. Effect of ionic strength on the acoustic generation of nitrite, nitrate and hydrogen peroxide. *Ultrason. Sonochem.* **6**, 141–148 (1999).
10. Mead, E. L., Sutherland, R. G. & Verrall, R. E. The effect of ultrasound on water in the presence of dissolved gases. *Can. J. Chem.* **54**, 1114–1120 (1976).
11. Bose, S., Mofidfar, M. & Zare, R. N. Direct conversion of N_2 and air to nitric acid in gas–water microbubbles. *J. Am. Chem. Soc.* **146**, 27964–27971 (2024).
12. Crum, L. A., Mason, T. J., Reisse, J. L. & Suslick, K. S. *Sonochemistry and sonoluminescence*. Vol. 524 (Springer Science & Business Media, 1998).
13. Supeno & Kruus, P. Sonochemical formation of nitrate and nitrite in water. *Ultrason. Sonochem.* **7**, 109–113 (2000).
14. Yusuf, L. A. *et al.* Toward decentralized nitrogen fixation using pulsed ultrasound. *Cell Rep. Phys. Sci.*, 102662 (2025).
15. Humphreys, J., Lan, R. & Tao, S. Development and recent progress on ammonia synthesis catalysts for Haber–Bosch process. *Adv. Energ. Sust. Res.* **2**, 2000043 (2021).
16. Jennings, J. R. *Catalytic ammonia synthesis: fundamentals and practice.* (Springer Science & Business Media, 1991).
17. Ren, Y. *et al.* Strategies to suppress hydrogen evolution for highly selective electrocatalytic nitrogen reduction: challenges and perspectives. *Energ. Environ. Sci.* **14**, 1176–1193 (2021).
18. Qing, G. *et al.* Recent advances and challenges of electrocatalytic N_2 reduction to ammonia. *Chem. Rev.* **120**, 5437–5516 (2020).
19. Yang, B., Ding, W., Zhang, H. & Zhang, S. Recent progress in electrochemical synthesis of ammonia from nitrogen: strategies to improve the catalytic activity and selectivity. *Energ. Environ. Sci.* **14**, 672–687 (2021).
20. Tang, X. C. *et al.* Recent advances in photocatalytic nitrogen fixation based on two-dimensional materials. *ChemCatChem* **16**, e202401355 (2024).
21. Sakakura, T., Murakami, N., Takatsuji, Y. & Haruyama, T. Nitrogen fixation in a plasma/liquid interfacial reaction and its switching between reduction and oxidation. *J. Phys. Chem. C* **124**, 9401–9408 (2020).
22. Zhao, X. & Tian, Y. Sustainable nitrogen fixation by plasma-liquid interactions. *Cell Rep. Phys. Sci.* **4**, 101618 (2023).
23. Han, G.-F. *et al.* Mechanochemistry for ammonia synthesis under mild conditions. *Nat. Nanotechnol.* **16**, 325–330 (2021).
24. He, C. *et al.* Mechanochemical synthesis of ammonia employing H_2O as the proton source under room temperature and atmospheric pressure. *ACS Sustain. Chem. Eng.* **10**, 746–755 (2022).
25. Rouwenhorst, K. H., Jardali, F., Bogaerts, A. & Lefferts, L. From the Birkeland–Eyde process towards energy-efficient plasma-based NO_x synthesis: a techno-economic analysis. *Energ. Environ. Sci.* **14**, 2520–2534 (2021).
26. Angineni, J., Reddy, P. M. K., Anga, S. & Somaiah, P. V. Nitrogen fixation by simple gling arc plasma reactor at elevated pressure for synthesis of aqueous nitrogen fertilizer. *Plasma Process. Polym.* **22**, 2400209 (2025).

27. Chen, S. *et al.* Direct electroconversion of air to nitric acid under mild conditions. *Nat. Syn.* **3**, 76–84 (2024).
28. Wan, H., Bagger, A. & Rossmeisl, J. Limitations of electrochemical nitrogen oxidation toward nitrate. *J. Phys. Chem. L* **13**, 8928–8934 (2022).
29. Bradu, C., Kutasi, K., Magureanu, M., Puač, N. & Živković, S. Reactive nitrogen species in plasma-activated water: generation, chemistry and application in agriculture. *J. Phys. D Appl. Phys.* **53**, 223001 (2020).
30. Bruggeman, P. J. *et al.* Plasma–liquid interactions: a review and roadmap. *Plasma Sources Sci. T.* **25**, 053002 (2016).
31. Gromov, M. *et al.* Electrification of fertilizer production via plasma-based nitrogen fixation: a tutorial on fundamentals. *RSC Sustain.* **3**, 757–780 (2025).
